# Hail formation triggers rapid ash aggregation in volcanic plumes

**DOI:** 10.1038/ncomms8860

**Published:** 2015-08-03

**Authors:** Alexa R. Van Eaton, Larry G. Mastin, Michael Herzog, Hans F. Schwaiger, David J. Schneider, Kristi L. Wallace, Amanda B. Clarke

**Affiliations:** 1 David A. Johnston Cascades Volcano Observatory, US Geological Survey, Vancouver, Washington 98683, USA.; 2 School of Earth and Space Exploration, Arizona State University, Tempe, Arizona 85287, USA.; 3 Department of Geography, University of Cambridge, Cambridge CB2 3EN, UK.; 4 Alaska Volcano Observatory, US Geological Survey, Anchorage, Alaska 99508, USA.

## Abstract

During explosive eruptions, airborne particles collide and stick together, accelerating the fallout of volcanic ash and climate-forcing aerosols. This aggregation process remains a major source of uncertainty both in ash dispersal forecasting and interpretation of eruptions from the geological record. Here we illuminate the mechanisms and timescales of particle aggregation from a well-characterized ‘wet' eruption. The 2009 eruption of Redoubt Volcano, Alaska, incorporated water from the surface (in this case, a glacier), which is a common occurrence during explosive volcanism worldwide. Observations from C-band weather radar, fall deposits and numerical modelling demonstrate that hail-forming processes in the eruption plume triggered aggregation of ∼95% of the fine ash and stripped much of the erupted mass out of the atmosphere within 30 min. Based on these findings, we propose a mechanism of hail-like ash aggregation that contributes to the anomalously rapid fallout of fine ash and occurrence of concentrically layered aggregates in volcanic deposits.

## Abstract (web summary)

The behaviour of airborne fine ash during explosive volcanic eruptions is poorly understood. Here, the authors study hail formation during an eruption, proposing a mechanism of particle aggregation that leads to the fallout of fine ash and the occurrence of concentrically layered aggregates in volcanic deposits

## Graphical abstract

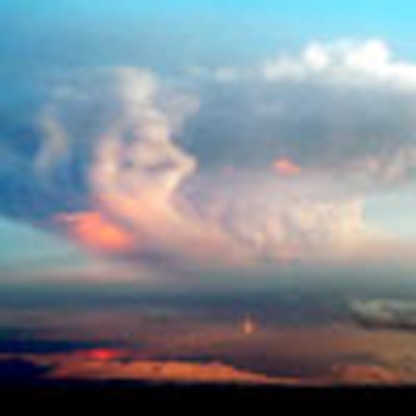

A number of explosive volcanic eruptions have been likened to ‘dirty thunderstorms' due to their powerful convective updrafts, elevated water contents and electrical activity^1^,^2^,^3^. All volcanic plumes contain some water originating from the magma, which is typically in the range of 2–7 wt.%. However, wet eruptions also incorporate water from external sources, by interacting with, for example, glaciers or aquifers. This water involvement is important because cloud microphysical processes—such as condensation and ice formation—impact volcanic plume development by scrubbing fine ash and sulfur from the atmosphere^4^,^5^,^6^,^7^,^8^. Despite recent observations that a major proportion of erupted fine ash never makes it into the downwind cloud, the actual mechanisms of near-source aggregation are poorly constrained, and therefore difficult to predict.

For decades, it has been proposed that spherical pellets of ash created during explosive eruptions (also referred to as accretionary lapilli) are akin to hailstones due to their similar size range, concentric structure and, sometimes, ice content^9^,^10^,^11^. For example, ash-laden hailstones fell during the Icelandic eruptions of Grímsvötn in 2011 (ref. 12) and Eyjafjallajökull in 2010 (refs 13, 14) and ‘ice-cold mudballs' splattered when they landed within 20 km of Mount St Helens on 18 May 1980 (ref. 15). The growth of dense, coherent ash aggregates requires liquid. Without it, particles are held together mainly by electrostatic forces, leading to loosely bound clusters that rarely survive impacts with the ground^14^,^16^,^17^. However, until now there have been no quantitative studies to examine how apparent similarities to hailstones relate to aggregate growth mechanisms in volcanic plumes. As a result of gaps in our fundamental understanding of ash aggregation, operational forecasting models currently lack physically based descriptions of the process, leading to overpredictions of ash dispersal and adverse effects on aircraft operations and related infrastructure^6^,^18^.

The eruption of Redoubt Volcano, Alaska, from March–April 2009 provides an illustrative and representative example of what happens when abundant external water is incorporated into a volcanic plume. In this case, the external water was sourced from a 100–200-m-thick glacier over the vent, which was partly destroyed by 3 weeks of explosive volcanic activity^19^. Many of the resulting water-rich ash plumes were detected in weather radar for only tens of minutes after explosions ended, and produced weak ash signals in satellite retrievals^20^,^21^. Rather than settling out of the atmosphere slowly and individually, the fine ash particles landed as frozen, spherical aggregates (‘volcanic hail') up to 10 mm in diameter^19^. Our analysis focuses on the well-documented period of activity from 12:30–13:00 UTC on 23 March 2009, referred to in the eruption sequence as explosive event 5. This event was chosen because the volcanic plume and downwind ash cloud were recorded in C-band Doppler radar and thermal infrared satellite^20^, and its fall deposits were readily distinguished in the field^19^. Moreover, it produced one of the most powerful volcanic lightning storms yet documented^22^. This study examines both direct and indirect evidence for the roles of water and ice in triggering the fallout of fine ash close to source. By combining observations from the fall deposits, weather radar, and three-dimensional (3D) simulation of the plume dynamics and microphysics, we establish new constraints on the mechanisms of ash aggregation, and effects on the long-distance dispersal of fine ash.

## Results

### Observations from the volcanic fall deposits

**Figure 1:**
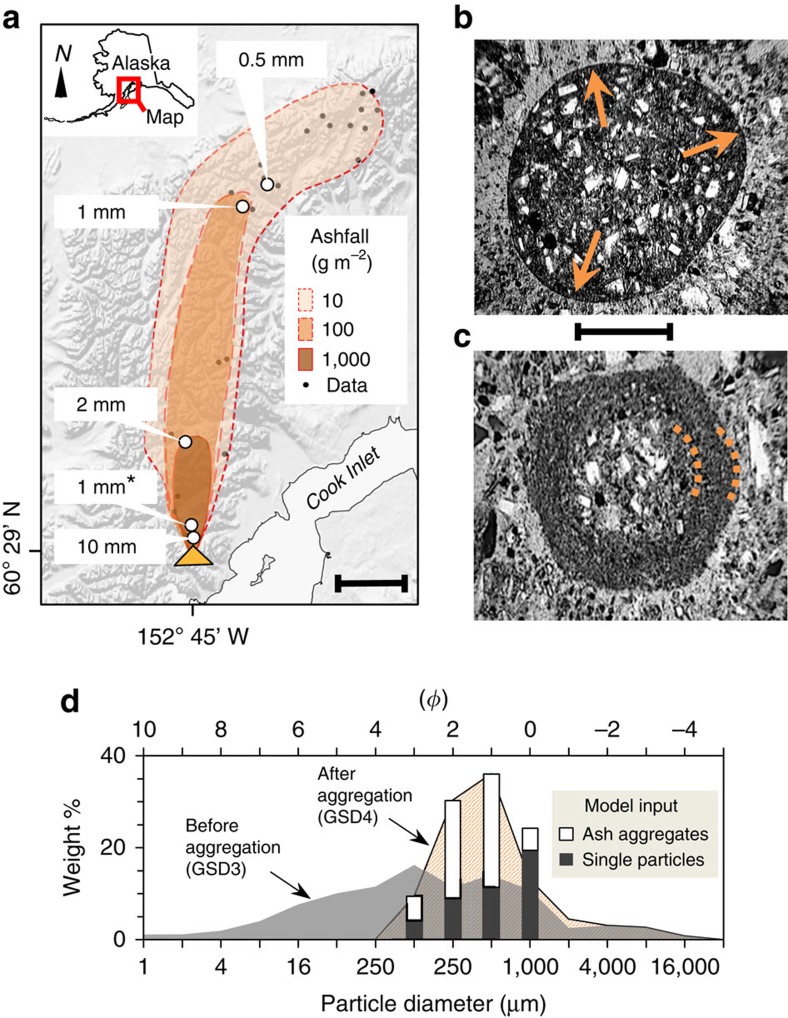
Physical observations of volcanic ash aggregation. Observations during the 2009 eruption of Redoubt Volcano in Alaska.(**a**) Extent of the mapped ashfall deposit^19^ from event 5 of the eruptive sequence. Scale bar, 50 km. Maximum diameters of ash aggregates indicated by white circles. ‘*' denotes two closely spaced sites. Yellow triangle shows location of Redoubt Volcano. (**b–c**) Thin sections of ash aggregates (plane-polarized light), which landed frozen 12 km from Redoubt Volcano. Scale bar, 1 mm. The frozen ash aggregates are commonly (**b**) unstructured with slight fining outward from the centre (arrows) or (**c**) contain concentric layers of ash particles. (**d**) Total grain-size distribution in the volcanic plume before and after aggregate formation. Data are binned into whole-*ϕ* intervals (where *ϕ*=−log_2_*D* in mm). Vertical bars indicate the eight volcanic tracers used in the ATHAM large-eddy simulation. Lower axis shows diameter in microns, upper axis in *ϕ*.

Field measurements and samples of ash aggregate fallout from Redoubt Volcano's event 5 were collected soon after the eruption, before melting destroyed their ice-rich structures^19^ (Fig. 1). Aggregate diameters increase markedly towards the volcanic source, with much smaller diameters persisting from 20 km to at least 229 km downwind (Fig. 1a; Supplementary Table 1). Photographs show that frozen ash aggregates and ash-laden hailstones fell at the same time, in the same location (Supplementary Fig. 1). The ash aggregates are internally massive to weakly layered, displaying concentric layers of ash particles (Fig. 1b,c). To estimate how aggregation increased the effective size of airborne particles, we compared the size distributions of three components from the fall deposit: (1) ash particles that make up aggregates, (2) the aggregates themselves, and (3) the erupted total before aggregation (See Methods and Supplementary Tables 1–3). Our analysis confirms that a highly size-selective aggregation process^14^ took place, preferentially stripping the smallest particles from the volcanic plume. From grain-size measurements, we estimate that >95% of the deposited fine ash (<250 μm) fell as larger aggregates, with only the remaining <5% left behind in the atmosphere as single particles (Supplementary Table 3). Figure 1d shows the airborne particle sizes before and after aggregation, taking into account the aggregates that formed and the single particles that were left behind (note: these components have different densities and fall velocities).

### 3D simulation of the eruption plume using ATHAM

**Figure 2:**
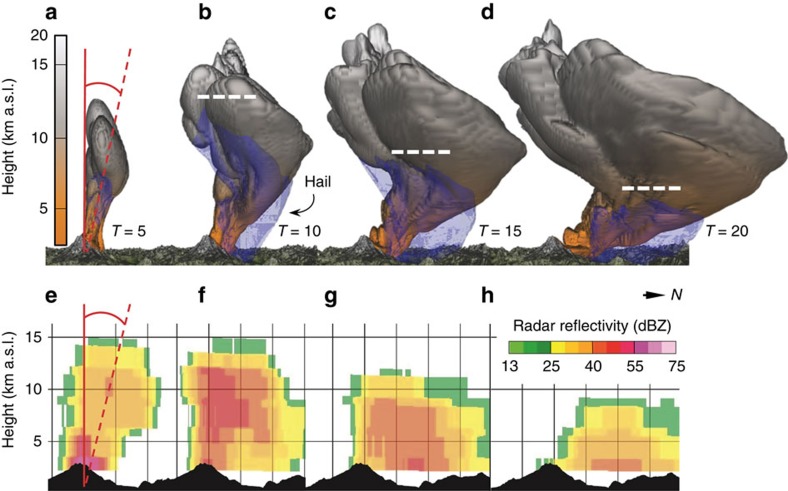
Comparison of 3D large-eddy simulation and measured radar reflectivity. (**a–d**) Modelled plume at *T*=5, 10, 15 and 20 min after eruption start. Note: eruption ends at *T*=6 min. Total volcanic particles (sum of single-particle and aggregate size bins) shown as an isosurface coloured by height and hail as a transparent blue isosurface, both at 1 mg m^−3^ concentration. Dashed white lines show maximum height of hail isosurface. Fallout of hail in the model corresponds to the rapid descent of radar echo tops from ref. 20, which is shown in **e–h** as north–south cross-sections at roughly equivalent time steps. Distance between vertical lines is 5 km.

**Figure 3:**
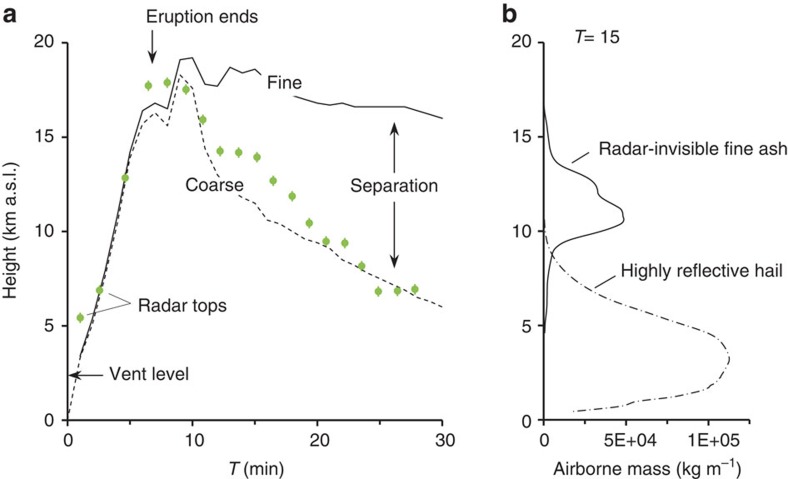
Gravitational separation of particles in the event 5 volcanic plume from Redoubt Volcano. (**a**) Time series showing maximum heights of the radar-detected plume (green circles) and modelled particles (lines). Mean radar heights derived from the highest-angle scan containing the cloud in plan view; error bars give maxima and minima. Solid line shows fine particles (≤250 μm); dashed shows coarse particles (≥500 μm), both at concentrations ≥10 mg m^−3^. (**b**) Modelled vertical distribution of mass at *T*=15 min, showing horizontally integrated mass of fine ash (solid) and hail (dot-dash).

**Figure 4:**
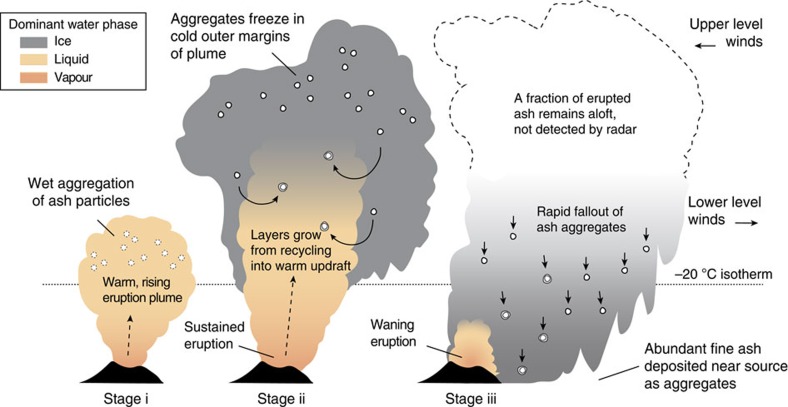
Schematic model showing hail-like growth of ash aggregates during explosive wet volcanism. Shaded colours indicate the dominant water phase coexisting and interacting with the airborne volcanic ash and gases. (i) Rapid coalescence of wet ash occurs in the presence of liquid water, within the warm updraft(s) of the rising, turbulent plume. (ii) Freezing takes place in the colder margins extending above the −20 **°**C isotherm of the background atmosphere (dotted line), which varies locally depending on geographical location and season. Fallout and turbulent re-entrainment may recycle frozen aggregates back into the warm core of the volcanic plume, leading to additional stages of wet growth and layering of ash particles. (iii) Weakening updrafts at the end of eruption lead to gravitational fallout of the larger aggregates, leaving behind a dilute cloud of fine-grained ash particles not detected by weather radar.

The frozen ash aggregates and ‘regular' hailstones originated near the eruptive source, indicated by their rapid size increase towards Redoubt Volcano (Fig. 1a). These observations strongly suggest that hail formation occurred directly within the volcanic plume. To test the physical plausibility of this process, we modelled event 5 using the Active Tracer High-resolution Atmospheric Model (ATHAM), which is a cloud-resolving large-eddy simulation for explosive eruptions^23^,^24^,^25^. The simulation was initialized from well-constrained volcanic and atmospheric characteristics of the event, including the post-aggregation grain-size distribution (GSD) derived from our measurements (Fig. 1d, Supplementary Fig. 2 and Supplementary Table 4). The 3D volcanic plume simulation generates high concentrations of hail, which fallout within 20 km of Redoubt Volcano (Fig. 2; Supplementary Movie 1). Locations and timescales of the hail formation show excellent agreement with C-band Doppler radar measurements and field deposits (Figs 2 and 3). Ten minutes after eruption initiation, the modelled plume reaches 19.2  km above sea level, matching the radar echo top (Fig. 4a). Shortly thereafter, coarse particles >500 μm, including hail and ash aggregates, begin to separate from the suspended mixture by gravitational fallout (Fig. 2b–d and Fig. 3b). At these same times, the radar shows a rapid decrease in plume height. We infer that the radar's sensitivity to the largest particles led to preferential detection of the region of ash aggregate formation and fallout^20^,^26^ (Figs 2e–h and 3b). Within half an hour, virtually all the hail has fallen out of the modelled cloud, consistent with the very low radar echo tops (Fig. 3a).

## Discussion

Combining our field observations and results from the mixed-phase cloud microphysics in ATHAM (Figs 2 and 3), we propose that the growth of coherent ash aggregates is ‘hail-like' in this case, both in terms of microphysics (involvement of liquid water and ice) and kinematics (cycling through turbulent updrafts). The process begins in a rising volcanic plume, sourced either directly over the vent (as shown in Fig. 4) or from ground-hugging pyroclastic density currents^27^,^28^. Liquid water condenses on ash particles, coalescing rapidly into unstructured pellets of wet ash. These warmer regions of the volcanic updraft contain abundant liquid water, favouring wet growth of ash aggregates similar to raindrop formation or glazing in hailstones^29^ (stage i, Fig. 4). Freezing subsequently occurs in the outer margins of the plume that entrain cold atmospheric air (stage ii, Fig. 4). Where temperatures fall below −20 **°**C, the sparse amounts of liquid water lead to dry growth—for example, by electrostatic attraction of particles^14^,^16^ or rapid freezing of supercooled droplets (riming^30^). Many of the frozen aggregates may fallout of the plume at this point, recording only a single pass through the warm, moist updraft. Alternatively, further layers of ash may be accreted during recapture into rising currents^31^ (stage iii, Fig. 4), modulated by turbulent eddies, changes in the eruption velocity at source, and bending or tilting of the volcanic plume by the ambient wind field. The layers in Redoubt Volcano's ash aggregates (Fig. 1b,c) are strong evidence for multiple passes through updrafts and downdrafts—this layering feature is commonly observed in aggregates from other deposits of wet eruptions in the geological record^11^,^27^,^28^,^32^. As the eruption wanes (stage iii, Fig. 4), updraft velocities can no longer support the ash aggregates and they settle out of the atmosphere, leaving behind only a small proportion of the original fine ash content (<5% in the case of Redoubt Volcano).

We suggest that hail-like growth of ash aggregates is most likely to occur when volcanic plumes: (1) ascend to atmospheric levels colder than −20 **°**C, where ash particles become effective ice nuclei^33^,^34^, (2) incorporate water from an external source (for example, a glacier), and (3) produce sustained updrafts, keeping particles aloft long enough to grow. These conditions were met during the eruptions of Grímsvötn in 2011, Eyjafjallajökull in 2010 and Mount St Helens in 1980. Indeed, volcanic hail was observed from each of these eruptions^12^,^13^,^14^,^15^.

It is worth noting that hail formation may not be constrained to eruptions in the mid- or high-latitudes. The −20 **°**C isotherm is typically near or below 10 km a.s.l., even in the tropics. This height is commonly reached by eruptions of moderate to high intensity (mass flux ≥10^6^–10^7^ kg s^−1^)^35^, suggesting that some amount of freezing and wet growth of aggregates may be a common occurrence during powerful, water-rich eruptions in general. However, there are certainly other scenarios in which hail formation is unlikely to occur, including eruptions that are too weak to rise higher than the atmospheric freezing level, or contain very little water from the magma, surface or atmosphere. In these cases, aggregation proceeds in the absence of ice—mainly by wet growth, electrostatic attraction or some combination of the two.

The hail-like growth process we have described played a key role in the overall extent of the fall deposits from the 2009 eruption of Redoubt Volcano. Using the volcanic ash transport and dispersal model Ash3d (refs 36, 37), we compared the fall deposit resulting from event 5 with and without an aggregated GSD (see Methods). In agreement with the mapped deposits, our results indicate that aggregation increased the near-source ashfall more than fivefold and reduced the long-distance ashfall by about half, defined by the areas receiving ≥1,000 and ≥10 g ash per m^2^, respectively (Supplementary Fig. 3). Aggregation scrubbed the volcanic plume of fine particles and shifted much of the erupted mass into lower regions of the atmosphere, causing early fallout and leaving behind a dilute cloud of remaining ash particles that escaped aggregation. This phenomenon has important implications for remote sensing during eruption response and ash hazards in general. To illustrate, our plume simulation (Figs 2 and 3) shows a cloud of fine-grained particles, including cloud ice, persisting at heights >10 km a.s.l. long after the fallout of larger hail and ash aggregates. The model results are in agreement with thermal infrared satellite images of an upper-level ash cloud drifting southeast >4 h after the event^20^,^21^. This upper-level cloud was not detected by the C-band radar, due to its small particle sizes and relatively dilute concentrations (ATHAM modelling suggests <250 μm and ≤4 g m^−3^, respectively). Based on this observation, we caution that volcanic plumes undergoing rapid aggregation may be visible in radar only briefly after the end of the eruption, while continuing to impact aviation at higher altitudes for hours to days.

Based on the present findings, we suggest that rapid, hail-like ash aggregation occurs directly within the plume arising from explosive, wet eruptions. Therefore, models of atmospheric dispersal for these scenarios may reasonably assume that ∼95% of the fine ash (<250 μm) is instantaneously converted into aggregates (Fig. 4). We have emphasized that water-rich eruptions are most strongly affected by this process, although further work is required to identify the range of eruption styles and plume water contents capable of triggering significant hail formation. Refining predictive models of long-distance ash transport thus requires a sink term in the proximal area to account for ash aggregation and related instabilities in the volcanic plume^23^,^38^,^39^,^40^, in addition to the formation of weaker ash aggregates that may grow in the distal cloud^41^. We conclude that rapid, hail-like growth of ash aggregates offers a compelling explanation for a diverse range of observations from volcanic plumes and their deposits, and provides a conceptual model to guide future development of ash dispersal forecasting.

## Methods

### Analysis of volcanic deposits

Detailed textural analysis was undertaken on volcanic ash aggregates from a well-preserved proximal location ∼12 km from Redoubt Volcano (Supplementary Fig. 1). The aggregates landed frozen and were archived in a freezer at the US Geological Survey (USGS) Alaska Volcano Observatory. The maximum aggregate diameter of ∼10 mm was recorded by Wallace *et al*.^19^. To examine these deposits in detail, we freeze dried them to remove ice without melting the aggregate structures^14^. Water content and frozen bulk density of individual aggregates (sample sizes *n*=8 and *n*=46, respectively) were also obtained by measuring the three principal axes, and weighing before and after oven drying. A representative size distribution of the intact, freeze-dried aggregates (*n*=1,182) was obtained from equivalent circular diameters of manually fitted ellipsoids using ImageJ analysis of 2D images. Aggregates <0.5 mm were not measured using this technique. A selection of representative aggregates was gently disaggregated for size analysis of the single, constituent particles using a Beckman–Coulter LS 13 320 Laser Diffraction Particle Size Analyzer. Data are provided in Supplementary Tables 1–3. Analyses assumed a refractive index of 1.56 and an absorption coefficient of 0.1 for andesitic ash.

### Calculating total GSD after aggregation

This study required an estimate of the total GSD in the volcanic plume after aggregation, which we refer to as GSD4. This represents the effective sizes of airborne particles during transport through the atmosphere. The calculation requires three key pieces of information, specifically the GSDs of: ‘single' particles that were incorporated into aggregates (GSD1); whole aggregates that formed during transport (GSD2); and single particles originally produced by the eruption before aggregation (GSD3).

For GSD1, we use the results of laser diffraction size analysis of ash aggregates collected 12 km from source (Supplementary Fig. 1), which were gently disaggregated before analysis. GSD2 required a mass-averaged size distribution of all aggregates in the deposit. To do this, we applied a method similar that of Murrow *et al*^42^. We inferred the spatial extent of three aggregate isopleths (lines of equal aggregate diameter) using the mapped distribution of deposit mass from ref. 19 from six widely spaced sites along the dispersal axis (12 to 229 km from source) where aggregate sizes were noted in the field (Supplementary Table 1). Ideally, isopleths would be based on observations at many more sites, but the speed with which frozen aggregates melt or are otherwise destroyed in harsh, high-altitude terrain precluded a larger data set. Nonetheless, our data set of ash aggregates is among the most detailed of any modern eruption yet documented. The GSD of aggregates contained within each of the three isopleths are assumed to follow a log-normal (Gaussian) distribution defined by a mean and s.d. from the measured values (that is, the maximum observed diameter was taken to represent the ∼99th percentile). The assumption of a log-normal distribution is based on the detailed analysis of the aggregates within 12 km of Redoubt Volcano, which gives an excellent match to the Gaussian fit (linear *R*^2^=0.91; see Supplementary Table 2). We use a minimum cut-off size of 125 μm for aggregate diameters, assuming that smaller clusters are of second-order importance to the overall volcanic plume dynamics. Deposit mass contained within each aggregate isopleth (Supplementary Table 1) was determined using the method of Fierstein and Nathenson^43^, assuming a single straight-line segment on a plot of log mass versus area^1/2^ (their Equation 13). Then, the mass in each aggregate size bin was multiplied by the fraction of total deposit mass contained within each isopleth, providing a mass-weighted aggregate size distribution that represents the overall sizes of whole aggregates produced during the 2009 event 5 eruption of Redoubt Volcano (Supplementary Table 3).

For GSD3, we use the distribution calculated by Mastin *et al*.^37^ (their Table 2), which employed a volume-weighted average of 32 grain-size analyses using the Voronoi tessellation technique^44^. These samples were disaggregated before analysis, and therefore represent the original total GSD of single particles before aggregation occurred. Once GSD1, 2 and 3 are established, it is possible to estimate how the aggregation process combines single particles from the original mixture into aggregates of known size distribution. It is important to note that aggregation is strongly size-selective, meaning that some of the particles are left out. We calculated the fraction of each single-particle size class (*ϕ*_s_) left behind after aggregation using the following:


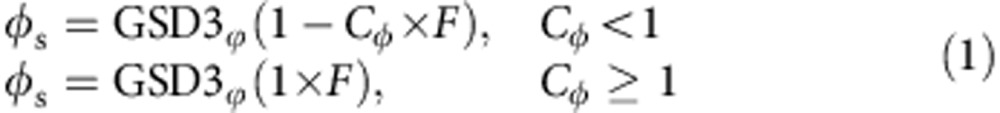


where GSD3_*ϕ*_ is the mass fraction in each size class of the original mixture, *F* is the fraction of total erupted mass undergoing collisions with other particles (a measure of time-integrated collision frequency^41^, here assumed to be 1), and *C*_*ϕ*_ is the aggregation coefficient, defined as the ratio of GSD1_*ϕ*_ to GSD3_*ϕ*_, which is different for each size class. The aggregation coefficient is related to a sticking efficiency^41^, accounting for the observation that finer particles are more likely to stick than coarser particles^14^,^17^. Note that under the assumption that 100% of the erupted mass is involved in this size-selective aggregation process (*F*=1), particles are completely converted into aggregates only when *C*_*ϕ*_≥1, that is, no single particles are left behind (*ϕ*_s_=0). Our analysis indicates that for Redoubt Volcano's event 5, the particles <0.125 mm in the mapped deposit were completely converted into aggregates, whereas a fraction of the particles between 0.125 and 1 mm were preferentially excluded from aggregation.

Then, the mass fraction moved into each aggregate size class (*ϕ*_a_) is calculated from the following:


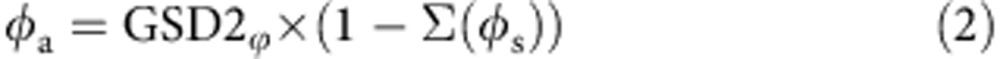


where GSD2_*ϕ*_ is the mass fraction in each class of the whole-aggregate size spectrum, and Σ(*ϕ*_s_) is the sum of all the size classes of single particles. Results of these calculations are shown in Supplementary Table 3. The effective size distribution after aggregation (GSD4) is then calculated by combining the single particles (*ϕ*_s_) and aggregates (*ϕ*_a_) in each size class.

### Large-eddy simulation of volcanic plume dynamics using ATHAM

ATHAM was used to simulate the 3D dynamics and microphysics of the event 5 eruption plume from Redoubt Volcano. ATHAM is a cloud-resolving, large-eddy numerical model that can handle dynamically and thermodynamically active tracers^23^,^24^,^25^. The basic information required to initialize the model includes: (1) a vertical profile of atmospheric temperature, wind velocity and relative humidity; and (2) a volcanic forcing at the lower model boundary, defined by a prescribed vertical flux of solid volcanic particles and gas of initially uniform temperature^25^ (in this case, 575 K). Volcanic source parameters were derived from detailed field observations (described in the following section). Atmospheric sounding data over Redoubt Volcano were interpolated from the 2.5-degree NCEP/NCAR Reanalysis 1 model^45^ (Supplementary Fig. 2). We used a stretched grid covering a domain of 100 × 100 × 30 km with 194 × 194 × 139 grid points. The maximum horizontal and vertical resolution of 50 m was centred at the volcano, stretching to 1.0 km (vertically) and 1.9 km (horizontally) at the model boundaries. Topography was interpolated onto the model grid from a 30-m resolution digital elevation model (Supplementary Table 4).

ATHAM's bulk microphysical scheme describes the exchange of mass and energy between water vapour and four hydrometeors: cloud water, cloud ice, rain and hail (see ref. 23 for full details). The hail tracer has a density of 700 kg m^−3^, which lies between that of true hail and lower-density graupel. There are 19 microphysical processes incorporated into the model, including condensation, evaporation, freezing, melting, sublimation, deposition, autoconversion and accretion. Hail growth is modelled by collection and freezing of rain and cloud water, and by deposition of water vapour. Fall velocities of hail and rain are taken from their volume mean radii, derived from Marshall–Palmer distributions, which depend on the mass concentration of each tracer^23^. In contrast, the radii of cloud water and cloud ice are prescribed and assumed to be monodispersed. A simplification of the bulk microphysical scheme is that it does not include interactions between hydrometeors and volcanic particles^23^, meaning they can coexist, but not combine. Therefore, processes of heterogeneous ice nucleation and ash removal by precipitation are not explicitly resolved in the model. To minimize these limitations, we do the following: (1) enable temperature-dependent statistical freezing of supercooled water beginning at 0 °C (ref. 23), which is reasonable given that contact freezing of liquid droplets is initiated by ice particles at all temperature <0 °C; and (2) use a coarser size distribution of volcanic particles representing the effective sizes after aggregation, based on measurements undertaken for this study (GSD4, Supplementary Table 3). Therefore, our simplifying assumptions are adequate for examining microphysical features in the volcanic plume, and identifying the regions in which hail-forming processes are thermodynamically favourable.

### Constraints on volcanic source parameters

Eruptive event 5 from Redoubt Volcano was exceptionally well-monitored by the USGS Alaska Volcano Observatory and partners, providing constraints on many of the eruptive properties required for modelling. The most sensitive inputs in our large-eddy simulation were related to eruption rate, including total erupted mass, eruption duration and initial velocity, followed by plume composition and plume temperature. Constraints on these key parameters are detailed below and summarized in Supplementary Table 4.

Erupted mass was calculated from fall deposit mapping by Wallace *et al*.^19^ as 4.4 × 10^9^ kg. We note that this value is based on the mass of oven-dried samples, and therefore does not include any of the water or ice involved in the eruption. The onset of eruption was detected by broadband seismometer at 12:30:21 UTC. Duration was inferred from the USGS C-band radar reflectivity, which showed that maximum reflectivity at the volcanic source (>50–55 dBz) lasted until ∼12:36:43 UTC^20^. We therefore assume that most of the erupted mass was injected into the atmosphere during these initial 6 min, which is consistent with ash dispersal modelling^37^ suggesting an eruption duration ≤10 min.

The constant initial velocity of the plume (19 m s^−1^) is taken from the radar-detected rise rate from the study by Schneider and Hoblitt^20^ during the first 1–2 min of eruption (15–20 m s^−1^). This somewhat slow ascent in the early stages of eruption suggest the plume was not simply a vertically directed blast; there may have been a laterally or radially directed component related to partial destruction of the lava dome, before convective ascent took over^7^. The exceptional lahar volume produced during this time is also consistent with mobilization of glacial ice in the Drift River valley by pyroclastic density currents of limited extent^46^.

Plume composition is described in our model by the mass fraction of volcanic particles and gases (mainly water vapour with minor SO_2_). The relative contributions of eight volcanic particle tracers and their densities were derived from textural analysis of the aggregated deposits, as described in the previous section (Supplementary Table 4). The amount of water vapour in the volcanic plume is an important consideration in this case, due to extensive interaction with the summit glacier. This is demonstrated, for example, by centimetre-sized glacier fragments blasted over 11 km from source during event 5 (ref. 19), and production of one of the most voluminous, ice-rich lahars ever documented^46^. Based on experimental studies of ash aggregation^14^, we infer that the eruption plume initially contained ∼20 wt.% water to produce the coherent aggregate structures observed. This value is consistent with measurements of water content from eight individual, frozen ash aggregates conducted for this study, which ranged from 17 to 29 wt.%. Therefore, we initialized the model with 3 wt.% water sourced from the magma^47^, and an additional 16.5 wt.% water incorporated from the glacier. We also included 0.5 wt.% SO_2_ gas as a non-reactive, bulk tracer to account for all other magmatic volatiles. The initial temperature of the volcanic plume was calculated assuming that magma-water mixing cooled the erupted mixture during thermal equilibration at constant pressure^48^, using a specific heat of magma of 1,000 J kg^−1^ K^−1^. We assumed that the erupting mixture (solid particles plus magmatic gas) was initially at 910 °C (ref. 47) before mixing with 16.5 wt.% liquid water at 0 °C, resulting in an equilibrated plume temperature of ∼300 °C.

Based on these considerations, we initialized the ATHAM model with a constant eruption rate of 1.53 × 10^7^ kg s^−1^ of volcanic particles and gases (1.22 × 10^7^ kg s^−1^, if only volcanic particles are included). Given the prescribed eruption duration of 6 min, our simulation erupts a mass of volcanic particles that is within 1% of the mapped value. The expanded plume diameter after equilibration to ambient pressure was adjusted to match the above constraints—however, our chosen diameter of 840 m is reasonable given that the Redoubt Volcano's summit amphitheatre (crater) has a diameter of nearly 2 km.

### Simulation of ash cloud dispersal using Ash3d

We used the Eulerian volcanic ash transport and dispersion model Ash3d (ref. 36), employing a model domain of 700 × 700 × 20 km and constant grid resolution of 5 km. Rather than resolving the near-source dynamics of the volcanic plume, Ash3d specializes in long-range transport of ash particles in a time-changing, 3D wind field. Ash3d was not coupled to ATHAM for this study. To examine how ash dispersal from event 5 of Redoubt Volcano would have been different if aggregation had not occurred, we initialized an Ash3d simulation using the total GSD before and after aggregation (GSD3 and GSD4, Supplementary Table 3) and other input parameters from ref. 37 (including maximum plume height of 15 km and a vertical distribution of mass defined by a Suzuki distribution with a *k*-constant of 4). The wind field was derived from the 32 km North American Regional Reanalysis data set^49^.

## Additional information

**How to cite this article:** Van Eaton, A. R. *et al*. Hail formation triggers rapid ash aggregation in volcanic plumes. *Nat. Commun*. 6:7860 doi: 10.1038/ncomms8860 (2015).

## Supplementary Material

Supplementary Information Supplementary Figures 1-3 and Supplementary Tables 1-4

Supplementary Movie 1 As in Figure 2, total volcanic particles (sum of single particles and ash aggregate size bins) are shown as an isosurface colored by height, and hail as a transparent blue isosurface. Both isosurfaces represent 1 mg m-3 concentration. View is from the east. Topography is from the 30 m-resolution digital elevation model (ASTER GDEM V2).
