# Supplementary material for: Hail formation triggers rapid ash aggregation in volcanic plumes
Source: Nat Commun. 2015 Aug 3;6:7860. doi: 10.1038/ncomms8860 (PMC4532834; doi:10.1038/ncomms8860)
Supplement: Supplementary Information — Supplementary Figures 1-3 and Supplementary Tables 1-4 [file ncomms8860-s1.pdf]

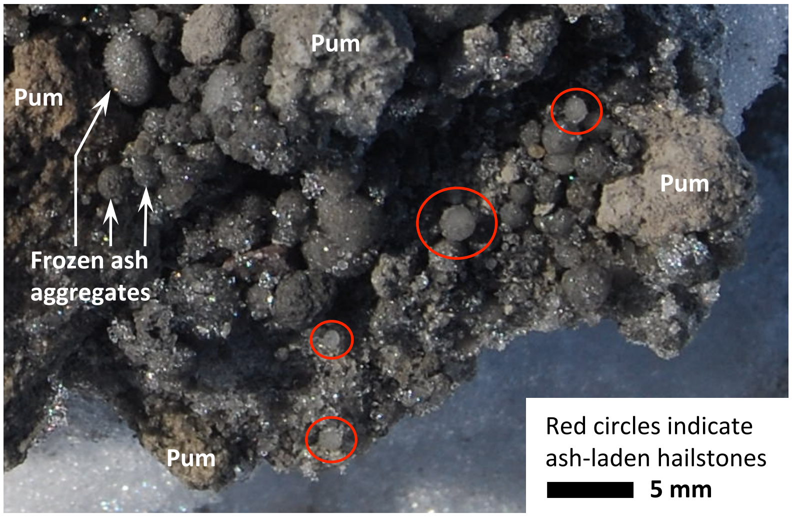

Supplementary Fig. 1. Photograph of frozen ash aggregates and ash-laden hailstones (red circles) that fell from the Redoubt event 5 volcanic plume. Location is approximately 12 km north of Redoubt Volcano at Juergen's Hut (see Supplementary Table 1 for grid reference). Examples of the frozen ash aggregates are indicated by white arrows; Pum = pumice clasts from the overlying deposits of event 6 in the eruptive sequence. Photo taken by Kate Bull on 21 April 2009. The size distribution of whole, intact aggregates from this site was obtained by image analysis (Supplementary Table 2). The maximum aggregate diameter of 10 mm was measured in the field by Wallace et al. (2013).

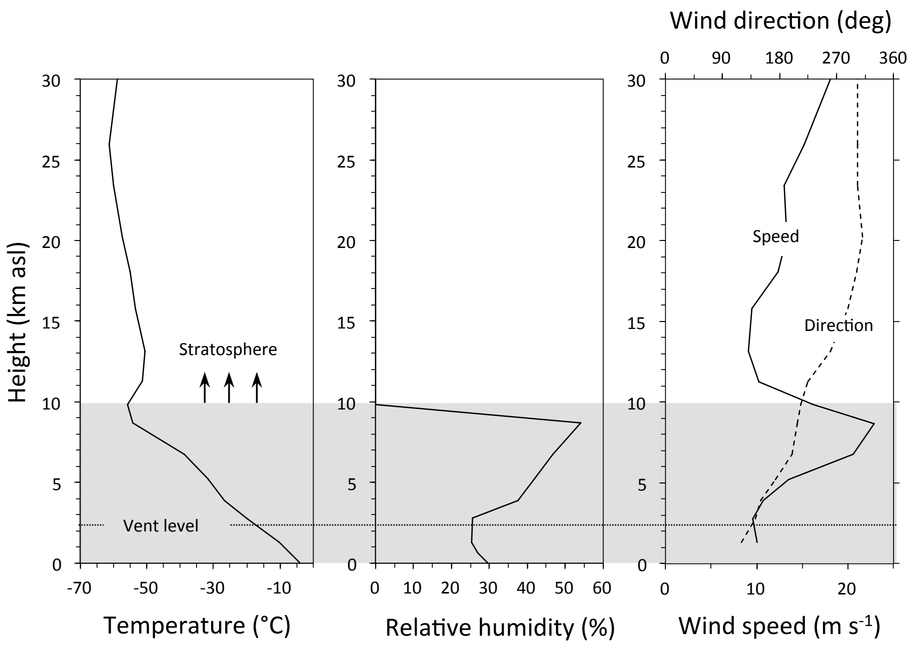

Supplementary Fig. 2. Meteorological inputs for the large-eddy simulation using ATHAM. Note the below-freezing surface temperatures, cold point tropopause located at approximately 10 km above sea level (indicated by gray shading), and variable wind speed and direction with height. Tropospheric winds blow mainly from the south or southeast, while upper level winds (>13 km asl) blow from the west or northwest. Sounding data were interpolated over Redoubt Volcano from the 2.5-degree NCEP/NCAR Reanalysis 1 model. Dotted line indicates the height of the volcanic vent.

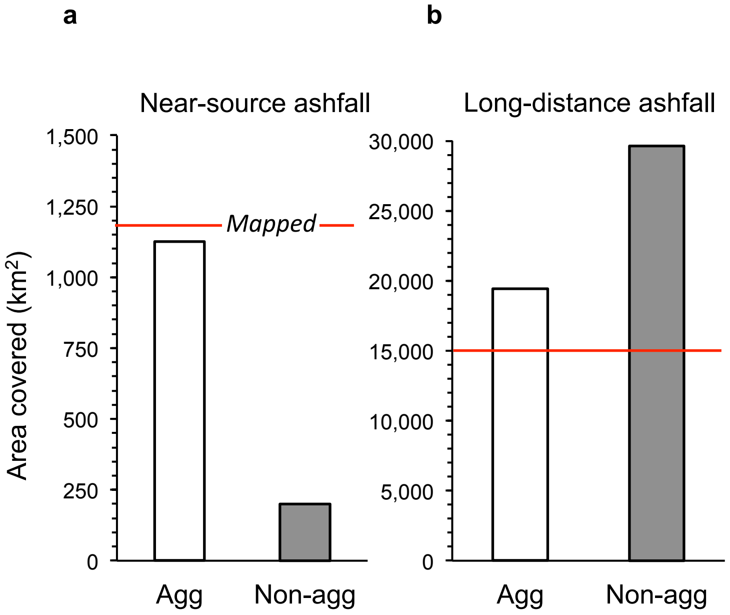

Supplementary Fig. 3. Comparison of ash dispersal from the Redoubt event 5 eruption modeled with and without aggregation. **a**, Area covered by near-source ashfall defined as receiving ≥1,000 g m^{-2}. **b**, Area covered by long-distance ashfall defined as receiving ≥10 g m^{-2}. Red lines show the mapped field values from Wallace et al. (2013). For the simulation neglecting aggregation (Non-agg), the dispersal model Ash3d was initialized with the total grain size distribution before aggregation, which contains single particles only (see GSD3 in Fig. 1d and Supplementary Table 3). The aggregated case (Agg) used the total grain size distribution after aggregation, which includes ash aggregates and the single particles excluded from aggregation (see GSD4 in Fig. 1d and Supplementary Table 3). Note that neglecting aggregation overpredicts the long-distance ashfall by a factor of approximately 2, and underpredicts the near-source ashfall by more than a factor of 5.

Supplementary Table 1. Aggregate diameters (*D*), sample sites, and isopleth data for total GSD of aggregates.

Sample ID	Distance from source (km)	Maximum *D* (mm)	Method	Latitude*	Longitude
09RDKLW002C	12	10.0	Field estimate; image analysis	60.5906015	-152.805779
09RDKLW068D	18	1.0	Field estimate: 0.5-1.0 mm	60.6504625	-152.764311
09RDKLW039D	19	1.0	Scaled field photos	60.6561919	-152.774660
09RDKLW041D	28	2.0	Scaled field photos	60.7307185	-152.810539
09RDJRS301A	213	1.0	Field estimate: 0.5-1.0 mm	62.3984300	-152.506230
09RDJRS307A	229	0.5	Field estimate: 0.5 mm	62.5347100	-152.211900

Aggregate isopleth	Mean aggregate *D* (mm)†	Standard deviation (mm)	% of total deposit mass contained in each isopleth	Isopleth area (km^2)
1st - Proximal‡	1.75	0.74	9.2	93.1
2nd - Medial	0.50	0.20	83.7	6,298.4
3rd - Distal	0.25	0.10	7.0	15,041.1

*Latitude and longitude reported in decimal degrees using the NAD1983 projection.

†Aggregate mean and standard deviation define the normal distribution for each isopleth.

‡Mean and standard deviation for 1st isopleth determined from image analysis (sample size n=1,182).

Supplementary Table 2. Calculation of the size distribution of whole aggregates (GSD2) for Redoubt event 5.

Size bins		Aggregate size distribution in each isopleth (% of total)				Mass-weighted GSD2*	
Diameter (ϕ)	Diameter (mm)	1st isopleth Measured distribution	1st isopleth Gaussian distribution	2nd isopleth Gaussian distribution	3rd isopleth Gaussian distribution	(%)	Normalized to 100%
3.00	0.125	-	0.12	0.91	4.98	1.12	1.16
2.75	0.149	-	0.15	1.36	7.66	1.68	1.75
2.50	0.177	-	0.19	2.07	11.34	2.54	2.63
2.25	0.210	-	0.26	3.20	15.46	3.78	3.92
2.00	0.250	-	0.34	4.98	18.19	5.47	5.67
1.75	0.297	-	0.47	7.66	16.79	7.63	7.91
1.50	0.354	-	0.66	11.34	10.61	10.29	10.67
1.25	0.420	-	0.93	15.46	3.79	13.29	13.77
1.00	0.500	0.08	1.35	18.19	0.59	15.39	15.95
0.75	0.595	0.25	2.00	16.79	0.03	14.24	14.76
0.50	0.707	1.10	3.02	10.61	0.00	9.15	9.49
0.25	0.841	1.61	4.57	3.79	0.00	3.60	3.73
0.00	1.000	5.75	6.89	0.59	0.00	1.13	1.17
-0.25	1.189	9.72	10.10	0.03	0.00	0.95	0.99
-0.50	1.414	17.75	13.88	0.00	0.00	1.28	1.32
-0.75	1.682	21.13	16.96	0.00	0.00	1.56	1.62
-1.00	2.000	16.65	17.03	0.00	0.00	1.57	1.62
-1.25	2.378	10.65	12.53	0.00	0.00	1.15	1.19
-1.50	2.828	7.86	5.75	0.00	0.00	0.53	0.55
-1.75	3.364	3.89	1.32	0.00	0.00	0.12	0.13
-2.00	4.000	1.86	0.11	0.00	0.00	0.01	0.01
-2.25	4.757	1.10	0.00	0.00	0.00	0.00	0.00
-2.50	5.657	0.25	0.00	0.00	0.00	0.00	0.00
-2.75	6.727	0.25	0.00	0.00	0.00	0.00	0.00
-3.00	8.000	0.08	0.00	0.00	0.00	0.00	0.00
-3.25	9.514	0.00	0.00	0.00	0.00	0.00	0.00
-3.50	11.314	0.00	0.00	0.00	0.00	0.00	0.00
-3.75	13.454	0.00	0.00	0.00	0.00	0.00	0.00
-4.00	16.000	0.00	0.00	0.00	0.00	0.00	0.00
-4.25	19.027	0.00	0.00	0.00	0.00	0.00	0.00
-4.50	22.627	0.00	0.00	0.00	0.00	0.00	0.00
-4.75	26.909	0.00	0.00	0.00	0.00	0.00	0.00
-5.00	32.000	0.00	0.00	0.00	0.00	0.00	0.00
	Totals	100.00	98.63	96.96	89.44	96.49	100.00

*Data are normalized to 100% assuming a minimum cutoff size of 125 microns for aggregates.

Supplementary Table 3. Grain size distributions (GSD) of eruption products from Redoubt Volcano's event 5, as % in each size bin.

Size bins		Aggregate characteristics		Total before aggregation	Total after aggregation		
Diameter (ϕ)	Diameter (mm)	GSD1* Single particles in aggregates	GSD2 † Whole aggregate diameters	GSD3 ‡ Total single particles	GSD4 § Total particles and aggregates	% from single particles	% from whole aggregates
>10	<0.001	1.87	0.00	1.12	0.00	0.00	0.00
10	0.001	3.33	0.00	1.12	0.00	0.00	0.00
9	0.002	4.80	0.00	1.15	0.00	0.00	0.00
8	0.004	7.24	0.00	1.93	0.00	0.00	0.00
7	0.008	11.93	0.00	4.01	0.00	0.00	0.00
6	0.016	17.36	0.00	7.67	0.00	0.00	0.00
5	0.031	18.63	0.00	10.08	0.00	0.00	0.00
4	0.063	17.37	0.00	11.54	0.00	0.00	0.00
3	0.125	12.12	9.46	16.26	9.44	4.14	5.30
2	0.250	2.25	38.02	11.20	30.27	8.95	21.32
1	0.500	2.54	43.92	13.96	36.05	11.42	24.63
0	1.000	0.55	5.10	10.82	13.13	10.27	2.86
-1	2.000	0.00	3.49	2.55	4.51	2.55	1.96
-2	4.000	0.00	0.01	3.06	3.07	3.06	0.01
-3	8.000	0.00	0.00	2.67	2.67	2.67	0.00
-4	16.000	0.00	0.00	0.83	0.83	0.83	0.00
-5	32.000	0.00	0.00	0.04	0.04	0.04	0.00
	Totals	100.00	100.00	100.00	100.00	43.93	56.07

**Percent total erupted mass excluded from aggregation**	**43.93**
**Fine ash (<250 microns) excluded from aggregation**	**4.14**

* Taken as the average of three laser diffraction grain size analyses of (gently crushed) aggregates 12 km from source.

† From mass-weighted average of aggregate isopleths shown in Supplementary Table 2.

‡ Voronoi volume-weighted average of 32 analyses from disaggregated, bulk deposit samples (after Mastin et al. 2013)

§ Sum of single particles and aggregates, as described in Supplementary Methods (Equations 1-2).

Supplementary Table 4. Input parameters for 3D large-eddy simulation using ATHAM.

Tracer description	Specific heat, C_p ($J\ kg^{-1}\ K^{-1}$)	Particle density ($kg\ m^{-3}$)	Particle diameter (µm)	Percent of erupted mix
Gaseous tracers				
Water vapor*	1,870	n/a	n/a	19.5
Sulfur dioxide	1,870	n/a	n/a	0.5
Hydrometeor tracers				
Cloud water	4,183	1,000	20	0.0
Rain	4,183	1,000	M-P dist.†	0.0
Cloud ice	2,103	917	20	0.0
Hail/graupel ‡	2,103	700	M-P dist.	0.0
Volcanic particle tracers				
125 µm glass	1,100	2,500	125	3.3
250 µm glass §	1,100	1,083	250	7.2
500 µm pumice	1,100	800	500	9.1
1 mm pumice	1,100	800	1,000	15.5
125 µm aggregates ♯	1,100	1,600	125	4.2
250 µm aggregates	1,100	1,600	250	17.1
500 µm aggregates	1,100	1,600	500	19.7
1 mm aggregates	1,100	1,600	1,000	3.9
Volcanic and topographic inputs ¶				
Vent position (degrees lat, lon)	60.4886, -152.7613			
Vent height (m above sea level)	2,300			
Eruption start time	23 Mar 2009 12:30:00 UTC			
Eruption duration (s)	360			
Model spinup before eruption (s)	300			
Exit velocity ($m\ s^{-1}$)	19			
Exit temperature (K)	575			
Total eruption rate ($kg\ s^{-1}$)	1.5E7			
Erupted mass of solids (kg)	4.4E9			
Diameter of expanded plume, m	846			
Digital Elevation Model (DEM)**	ASTER GDEM V2			
Grid properties	x	y	z	
Extent of spatial domain (km)	100	100	30	
Number of grid points	194	194	139	
Number of processors	8	8	n/a	
Finest resolution (m)	50	50	50	
Coarsest resolution (m)	1,900	1,900	1,000	

* Amount derived from the minimum wt.% water of frozen ash aggregates, plus 3 wt.% magmatic water.

† Mean size described by a Marshall-Palmer distribution, which is a function of tracer concentration.

‡ Tracer has a density between that of true hail and lower-density graupel (i.e., snow pellets).

§ Density based on the averaged componentry of Wallace et al. (2013)

♯ Densities taken from the average density of (frozen) ash aggregates from Redoubt Event 5.

¶ Refer to Supplementary Methods for constraints on eruption source parameters.

** Original DEM resolution of 30 m was resampled to the resolution of the model grid.
